# Sex-dependent transcriptional and epigenetic regulation of neutrophil inflammatory programs in COPD

**DOI:** 10.3389/fimmu.2026.1824596

**Published:** 2026-05-28

**Authors:** Barbara Mariotti, Sara Gasperini, Chiara Bracaglia, Carlo Frigenti, Giulia Sartori, Claudia di Chiara, Francesca Sangiovanni, Ernesto Crisafulli, Flavia Bazzoni

**Affiliations:** 1 Department of Medicine, Division of General Pathology, University of Verona, Verona, Italy; 2 Department of Medicine, Respiratory Medicine Unit, University of Verona and Azienda Ospedaliera Universitaria Integrata of Verona, Verona, Italy

**Keywords:** COPD, epigenomic, neutrophil, sex, transcriptomic

## Abstract

**Introduction:**

Sex differences strongly influence immune responses and susceptibility to inflammatory diseases, yet how biological sex shapes immune regulatory mechanisms in chronic obstructive pulmonary disease (COPD) remains poorly understood. Neutrophils are key drivers of COPD pathogenesis, but whether biological sex shapes their inflammatory programming has not been systematically investigated.

**Methods:**

Here we integrated transcriptomic and epigenomic profiling of circulating neutrophils from male and female COPD patients to define sex-dependent regulatory programs in innate immune cells.

**Results:**

Unsupervised analyses revealed that sex represents a major source of transcriptional variation in neutrophils. Although COPD induced a shared disease-associated transcriptional signature in both sexes, the magnitude and functional orientation of this response differed markedly. Male COPD neutrophils displayed robust enrichment of interferon signaling, cytokine-mediated pathways, and inflammatory networks. These transcriptional changes in male were accompanied by widespread H3K27ac enrichment at promoters and enhancers of inflammatory loci, and by elevated plasma levels of CXCL8, TNF-α, IFN-α, IFN-γ, and VEGF inflammatory mediators. In contrast, female neutrophils preferentially exhibited transcriptional programs related to autophagy and vesicle-mediated processes.

**Discussion:**

These findings define a model in which COPD elicits a shared neutrophil transcriptional framework that is amplified through sex-specific epigenetic and inflammatory feedback. This male-biased inflammatory reprogramming provides a mechanistic basis for sex differences in COPD immunopathology and highlights the importance of incorporating sex as a biological variable in the development of precision therapies for chronic inflammatory disease.

## 1 Introduction

Chronic obstructive pulmonary disease (COPD) is a progressive inflammatory condition characterized by irreversible airflow limitation and systemic immune dysregulation.

COPD is a biologically heterogeneous pathology, yet the determinants of this heterogeneity remain incompletely resolved. Among the factors contributing to this heterogeneity, sex has emerged as a fundamental axis shaping disease susceptibility, clinical expression, and progression. This recognition is relatively recent; historically, COPD was conceived as a predominantly male disease, a view that contributed to systematic under-recognition and misdiagnosis in women and delayed the appreciation of sex-specific disease biology (1).

However, COPD should no longer be regarded as a condition primarily affecting males, as its prevalence in females has risen to match that observed in males over the past decade. Despite a comparable incidence rate, a growing body of studies demonstrated that, for a comparable smoking exposure or other environmental insults, males and females display divergent phenotypic patterns, symptom presentation, clinical characteristics, lung function decline, exacerbation risk, comorbidities, prognosis, and treatment responses. These differences have been extensively synthesized in recent reviews (2, 3). Importantly, these disparities point toward underlying biological mechanisms, including sex hormones, epigenetic regulation, differences in lung development, aging, and, last but not least, immune modulation. In fact, COPD is characterized as a disease driven by chronic immune dysregulation, involving both innate and adaptive immunity, contributing not only to pulmonary damage but also to systemic effects. At the same time, a growing body of research shows that sex hormones and sex-specific biology significantly shape immune responses, influencing the immune responsiveness and the incidence of inflammatory diseases (4, 5).

In COPD, females exhibit higher plasma levels of IL-16 and adipokines, whereas males show elevated VEGF and altered T-cell subsets (6, 7). This sexual dimorphism suggests that immune activation and systemic inflammation are not only consequences of COPD but are also shaped by sex-dependent regulatory networks.

Within dysregulated innate immunity, neutrophils have been shown to be key drivers of COPD pathogenesis (8). They have been shown to accumulate in the lungs, releasing proteases and reactive oxygen species that contribute to airway damage and tissue remodeling, and to increase in circulation (9, 10). Using single-cell analyses, Kapellos and colleagues demonstrated that blood neutrophils are already increased in early-stage COPD (11). With regard to this issue, we recently provided evidence suggesting that sex is a significant factor in the dynamic changes in the number of circulating neutrophils in COPD patients (12). Specifically, we demonstrated that an increase in neutrophil counts is an early hallmark of COPD in males, but not in females. Additionally, recent molecular profiling has refined this view, showing that COPD blood neutrophils exist in transcriptionally distinct states characterized either by dysregulated cytokine signaling, phagocytosis, cell death pathways (11) or by a “poised” state rendering them more responsive to a second microbial challenge (13).

Beyond transcriptional alterations, accumulating evidence also implicates epigenetic mechanisms in COPD pathobiology. In particular, changes in DNA methylation patterns within circulating blood cells have been consistently associated with both disease status and lung function impairment (14, 15). Other than DNA methylation, histone remodeling through acetylation and methylation also critically regulates gene expression across several respiratory diseases, including COPD (16). However, very few studies have successfully identified the specific functional roles of these histone modifications in the context of COPD (16). Recently, we demonstrated that COPD neutrophils have altered H3K4me3 epigenetic profile and that these modifications are responsible for potentiating neutrophil pro-inflammatory response to microbial stimulation. Remarkably, the degree of H3K4me3 increases along with the disease severity (13). To date, no study addressed the role of H3K27ac, a marker of active transcription, on transcriptional reprogramming of COPD neutrophils. Importantly, no studies have investigated the role of sex in epigenetic modifications in COPD blood neutrophils.

Despite the well-established role of neutrophils in the pathogenesis of COPD, the influence of sex on the reprogramming of the transcriptional and epigenetic landscape that drives neutrophil dysfunction in COPD remains poorly characterized.

This study was designed to interrogate the sex-dependent transcriptional and epigenetic reprogramming of circulating neutrophils in COPD. By employing RNA-seq and ChIP-seq for H3K27ac marks, and deploying principal component and differential analysis, we sought to define how sex shapes the molecular phenotype of neutrophils in COPD, thereby informing both mechanistic understanding and clinical translation.

## 2 Materials and methods

### 2.1 Patients

COPD patients (n=36) and age- and sex-matched controls (n=35) were recruited at the Respiratory Medicine Unit of the Azienda Ospedaliera Universitaria Integrata of Verona. All participants provided written informed consent approved by the local ethics committee (protocol 42052/2015), in accordance with the Declaration of Helsinki, and met the following inclusion criteria: (i) older than 55 years old; (ii) not affected by infectious, inflammatory, autoimmune, lung (other than COPD), and neoplastic diseases or cancer history in the last three years; (iii) not under treatment with oral or systemic steroids; (iv) not under treatment with antibiotics. Lung function was tested according to international recommendations (17) as previously described (12). The main demographic and clinical data of the enrolled donors were summarized in **Supplementary Table 1**. **Supplementary Table 2** shows the main demographic and clinical data for male and female donors, separately.

### 2.2 RNA sequencing data analysis

RNA-seq data analyzed in this study were generated in a previously published work (13) from purified blood neutrophils of the enrolled COPD patients and age- and sex-matched controls. Granulocytes were isolated under endotoxin-free conditions from K_2_EDTA-anticoagulated whole blood by Ficoll-Paque PLUS density-gradient centrifugation, and neutrophils were immediately processed for RNA extraction and Chromatin Immunoprecipitation (ChIP). RNA-seq was performed using the QuantSeq 3′ mRNA-Seq Library Prep Kit (Lexogen), and raw data are publicly available in the GEO database (accession number GSE278400). In contrast to the original study, in which sex was included as a covariate, the present reanalysis was conducted in a sex-disaggregated manner. Gene expression was quantified using the fpm function implemented in DESeq2 (18) and expressed as fragments per million mapped reads (FPM); genes with an average FPM > 1 were considered expressed. Differentially expressed genes were identified according to the Wald test implemented in DESeq2. Genes modulated with a p-value< 0.05 were considered differentially expressed. We did not apply a threshold for the fold change. Smoking status has been considered as a covariate in the differential expression analysis. Gene Ontology (GO) term analysis was performed using the Gene Ontology Enrichment Analysis tool (https://geneontology.org/).

### 2.3 Chromatin immunoprecipitation sequencing

H3K27ac chromatin immunoprecipitation was performed on freshly isolated neutrophils from COPD patients (n=11) and control donors (n=12), as previously described (13). ChIP-seq libraries were prepared using the NEXTflex Rapid DNA Sequencing Bundle Illumina Compatible (Bioo Scientific, a PerkinElmer Company, Austin, TX, USA) according to the manufacturer’s protocol. Differential histone acetylation analysis was conducted in a sex-disaggregated manner using DESeq2 (18). Only histones modulated with a p-value<0.05 were considered differentially acetylated. Annotation and gene ontology (GO) term enrichment analysis of selected regions was performed with ChIPseeker (v 1.32.1). The annotation range around the transcriptional start site (tssRegion parameter of annotatePeaks function) was set to -3000,3000.

### 2.4 Hierarchical clustering and principal component analysis

Hierarchical clustering analysis of the transcriptional profile of circulating neutrophils was calculated based on the entire set of expressed genes using the Ward.D2 agglomerative algorithm on the Euclidean distance matrix. Principal Component Analysis (PCA) was performed using the PCAtools package in R. The top 1,000 most variable genes and the top 500 most variable acetylated regions have been analyzed. The optimal number of PC to analyze was determined according to the Elbow statistics.

### 2.5 Gene set variation analysis and gene set enrichment analysis

To analyze the expression of the previously published transcriptomic signature of COPD neutrophils (13) in the transcriptomic profile of neutrophils from male and female COPD patients, we used the Gene Set Variation Analysis (GSVA, v1.44.5) package in R. Variation of the COPD signature in the transcriptomic profile of neutrophils and control donors was calculated using the gsva function with method = “gsva”. Pathway enrichment and variation were analyzed using the Gene Set Enrichment Analysis (GSEA) tools (https://www.gsea-msigdb.org/). Enrichment of the pathways available in the Hallmark and Reactome repositories among the differentially expressed genes identified from RNA-seq analysis was calculated using the GSEA Preranked tool. Differentially expressed genes were ranked based on the log_2_(fold change) value obtained from DESeq2 analysis. Pathways with FDR q-value < 0.06 were considered enriched.

### 2.6 Ingenuity pathway analysis of upstream regulators

Upstream regulators of transcriptional reprogramming were identified using QIAGEN Ingenuity Pathway Analysis (IPA) software (QIAGEN Inc.). Briefly, the fold change of the differentially expressed genes was used as input for the Core analysis implemented in IPA software. Upstream regulators belonging to the cytokine, chemokine, and growth factor categories, with a p-value of overlap < 0.05 and an activation z-score > 1.5, were considered for subsequent analysis.

### 2.7 Quantification of plasma levels of CXCL8, IFN-α, IFN-γ, IL-1β, IL-17A, TNF-α, and VEGF

Plasma levels of CXCL8, IFN-α, IFN-γ, IL-1β, IL-17A, TNF-α, and VEGF were quantified using a specific ProQuantum Immunoassay Kit (ThermoFisher Scientific) according to the manufacturer’s instructions. Briefly, 3 μL of diluted plasma (1:1 in PBS) were further diluted in sample diluent (1:1) and then incubated in the presence of two oligonucleotide-conjugated analyte-specific antibodies for 1 h at room temperature. Next, ligation and qPCR were conducted in the presence of specific master mix, on the ViiA7 Real-time PCR System (ThermoFisher Scientific). Data were analyzed using the ProQuantum Software (ThermoFisher Scientific). Standard curves were calculated using the four-parameter logistic regression (4PL).

### 2.8 Statistics

Dataset distribution was evaluated using the Shapiro-Wilk normality test, and according to it, differences between two groups were analyzed using a non-parametric Mann-Whitney test or an independent t-test, as appropriate. Two-way ANOVA analysis followed by Tukey’s multiple comparison test evaluated differences between multiple groups. Correlations were analyzed using the Spearman statistic. Statistical significance was defined as a p-value<0.05. Statistical analysis was performed using GraphPad Prism version 8.0 (GraphPad Software Inc.) and R.

## 3 Results

### 3.1 Sex is the major determinant of neutrophil transcriptional profiles in COPD

**Figure 1:**
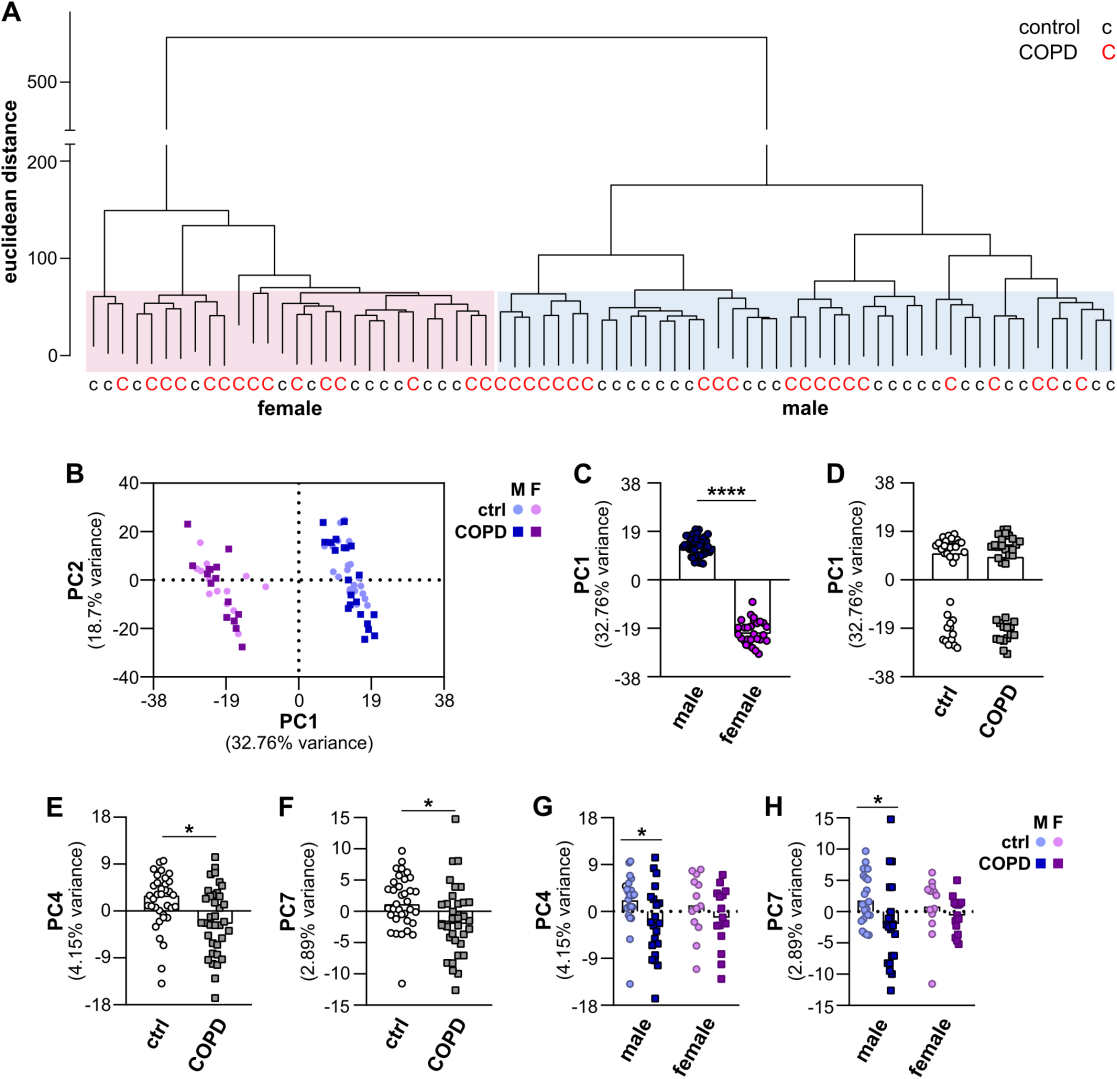
Sex influences the transcriptional profile of circulating neutrophils. **(A)** Hierarchical clustering dendrogram of the transcriptional profile of circulating neutrophils from COPD patients **(C)** and control donors (c). **(B)** Principal component analysis (PCA) biplot showing PC1 and PC2 computed from the 1,000 most variable genes expressed in neutrophils from COPD patients (squares) and control donors (circles). The percentage of variance explained by each principal component is indicated. Male donors are shown in blue and female donors in pink. **(C–H)** Distribution of PC1, PC4, and PC7 scores in COPD patients and control donors **(D–F)** and in male and female subjects **(C, G, H)**. *p-value < 0.05; ****p-value<0.0001 determined by Mann-Whitney test.

To delineate the impact of sex on neutrophil transcriptome in COPD, RNA sequencing data from 36 patients and 35 matched control donors, previously published by our group (13) (GEO accession number GSE278400), were re-analyzed in a sex-disaggregated manner, as specified in Materials and methods. Donor characteristics are reported in **Supplementary Table 1**. Unsupervised hierarchical clustering analysis (HCA) of gene expression identified two distinct clusters, stratifying donors primarily by sex rather than disease status (**Figure 1A**). Principal component analysis (PCA) of the 1,000 most variable genes confirmed this observation, with PC1 (32.76% variance) clearly separating male and female samples (**Figures 1B, C**). In contrast, COPD status did not influence the global transcriptomic landscape (**Figure 1D**).

Given that sex emerged as the principal driver of transcriptional changes in an unsupervised analysis, we next sought to determine how COPD itself could influence, and to what extent, the transcriptional reprogramming. To this end, we explored disease-specific transcriptional variation by examining higher-order principal components (beyond PC1 and PC2) obtained from the PCA. The discriminatory capacity between COPD patients and control donors was assessed across the first nine principal components (PCs) of the transcriptomic dataset, as defined by the Elbow statistic (**Supplementary Figure 1A**). Among these, only PC4 (explaining 4.15% variance; **Figure 1E**) and PC7 (2.89% variance; **Figure 1F**) significantly differentiated COPD patients from controls (p-value < 0.05). Upon stratification of PC4 (**Figure 1G**) and PC7 (**Figure 1H**) data by sex, this effect was restricted to males, with no significant discrimination detected among females. No other PCs differentiated patients from controls, irrespective of sex (**Supplementary Figures 1B–M**).

### 3.2 Expression of inflammatory and interferon response-associated genes characterizes neutrophils from male COPD patients

Given that sex is a major driver of transcriptional differences in circulating neutrophils, we investigate whether it affects neutrophils from COPD patients compared with those from control donors. The activation and maturation state of circulating neutrophils were assessed by cytofluorimetric analysis of CD11b, CD11c, CD35, CD62L, and CD10. No sex-associated differences in the level of membrane-bound activation/maturation markers in neutrophils from COPD and controls were observed (**Supplementary Figures 2A, B**). In line with previous data (12), an increased number of circulating neutrophils was observed in male COPD patients compared to controls, but not in females (**Supplementary Figure 2C**).

**Figure 2:**
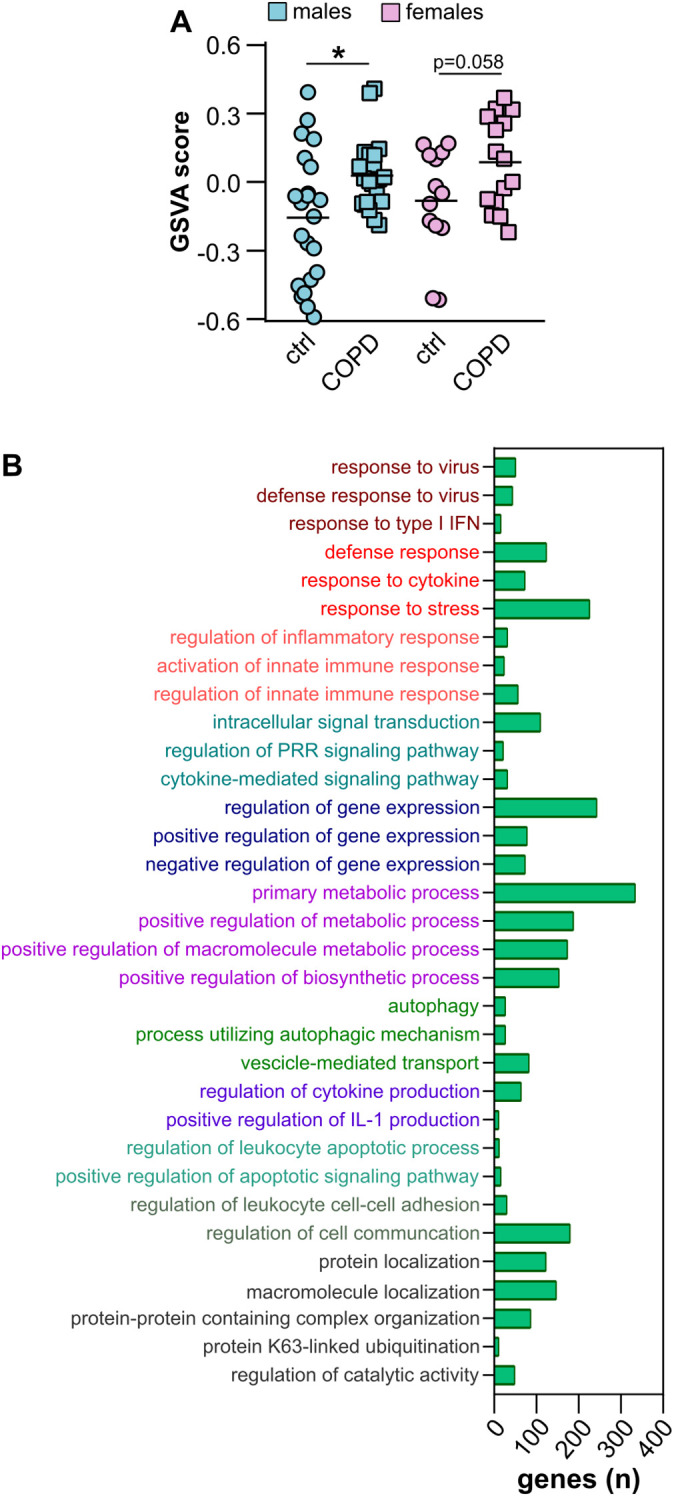
Characterization of the COPD-associated transcriptional signature. **(A)** Gene Set Variation Analysis (GSVA) of the COPD-associated transcriptional signature in neutrophils from male and female COPD patients and control donors. *p-value< 0.05 according to the Mann–Whitney test. **(B)** Gene Ontology (GO) term enrichment analysis of the COPD-associated signature. Bars indicate the number of differentially expressed genes (DEGs) associated with each GO term. Terms related to similar biological functions are shown in the same color.

In our previous study (13), sex was modeled as a confounding variable to account for its influence on gene expression, leading to the identification of a set of genes upregulated in neutrophils from COPD patients compared with controls (hereafter referred to as the COPD signature, **Supplementary Table 3**). In order to identify the relative contribution of sex in the COPD signature, gene set variation analysis (GSVA) was performed in male and female patients and controls. GSVA confirmed that the COPD signature was enriched in the transcriptomes of neutrophils from patients of both sexes. The enrichment was more pronounced in males (p-value<0.05) than in females (p-value= 0.058) (**Figure 2A**). Together, these findings indicate that the primary transcriptional alterations previously linked to COPD are preserved when sex is not included as a covariate, although the magnitude of their activation appears to be more prominent in males. GO enrichment analysis of sex-corrected transcriptomic dataset highlights biological processes involved in immune activation (response to virus, response to type I interferon (IFN), response to cytokine, regulation of inflammatory/innate immune responses), in intracellular signal transduction (regulation of PRR signaling pathway, cytokine-mediated signaling pathway), in regulation of gene expression and metabolic rewiring (regulation of biosynthetic and metabolic process) in autophagy regulation (autophagy, process utilizing autophagic mechanism, vesicle-mediated transport), and in leukocytes functions (IL-1 production, apoptotic process, leukocyte cell adhesion) (**Figure 2B**).

**Figure 3:**
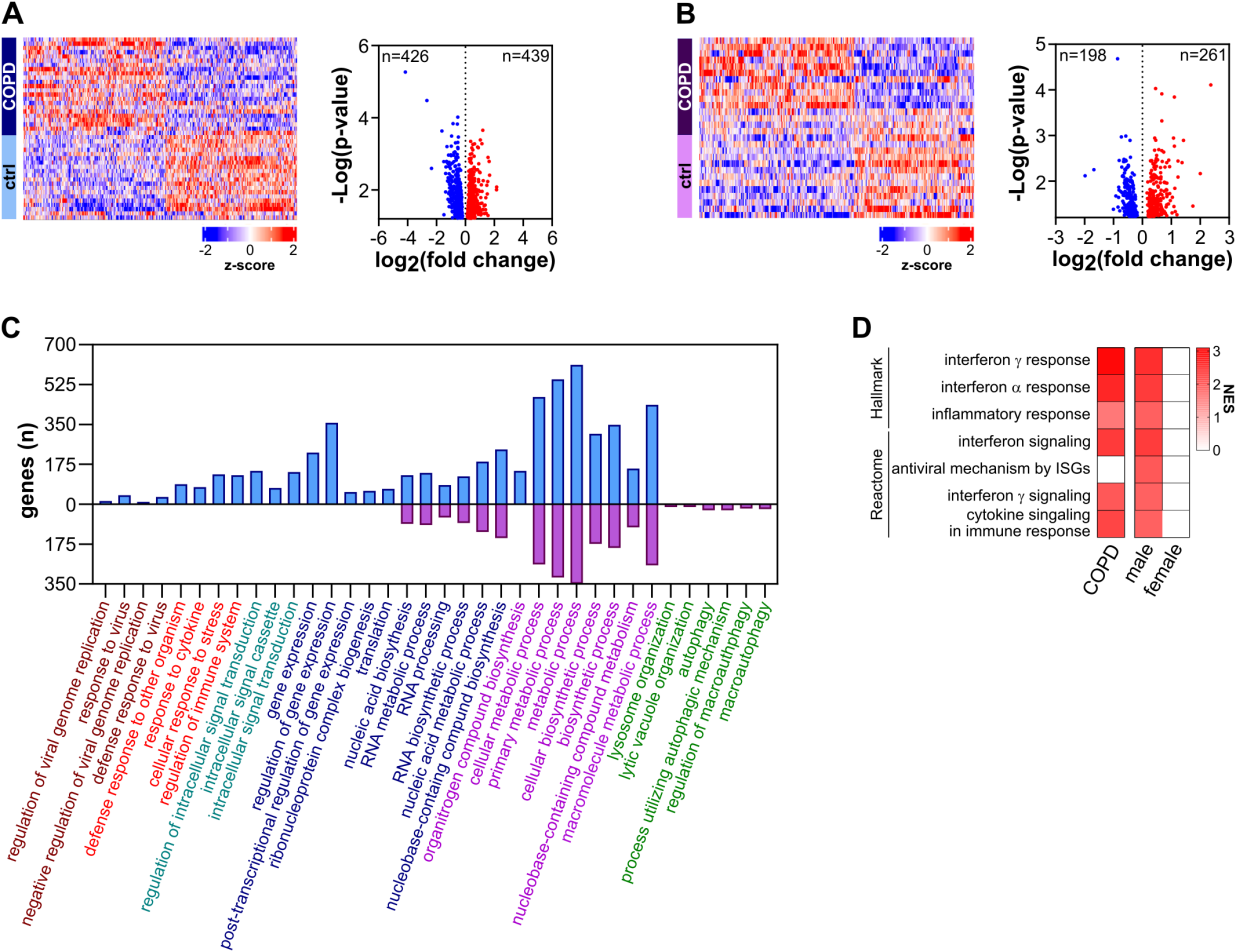
Sex-specific transcriptional remodeling of neutrophils in COPD. Heatmaps and volcano plot of the differentially expressed genes (DEGs) in neutrophils from male **(A)** and female **(B)** COPD patients compared to sex-matched controls. Heatmaps show the z-score of the Fragment per Million mapped reads (FPM). **(C)** GO term enrichment analysis of the 865 DEGs identified in male patients (blue bars) and the 459 DEGs identified in female patients (pink bars). Bars represent the number of DEGs associated with each GO term; related biological processes are indicated by the same color. **(D)** Gene Set Enrichment Analysis (GSEA) was performed on pathways from the Hallmark and Reactome repositories using genes differentially expressed in neutrophils from COPD patients compared with controls, analyzed either in a sex-corrected manner (COPD) or in a sex-disaggregated manner comparing male COPD patients versus male controls (male) and female COPD patients versus female controls (female). Heatmap colors represent the normalized enrichment score (NES) according to the indicated scale. NES values for non-significant enrichments were arbitrarily set to 0.

Differential gene expression (DGE) analysis performed in a sex-disaggregated manner identified 865 genes differentially expressed in male COPD patients compared to controls (**Figure 3A**), and 459 genes differentially expressed in female patients (**Figure 3B**). Notably, the overlap between male and female DEGs was minimal (81 genes, 6.52% of the total DEGs, not shown), underscoring distinct sex-specific responses.

Sex-disaggregated GO enrichment analysis reveals that the major biological processes identified in the sex-corrected analysis (**Figure 2B**) are preserved across sexes, but their relative contributions differ (**Figure 3C**). Both male and female COPD patients exhibited significant enrichment in biosynthetic and metabolic processes, indicating shared alterations in fundamental metabolic activity (**Figure 3C**). However, distinct sex-specific enrichment patterns were also evident. Transcriptional modulation in males primarily contributed to the enrichment of antiviral defense mechanisms, cytokine-mediated signaling, type I IFN response pathways, and broader immune system processes. In contrast, DEGs identified in female patients were predominantly associated with pathways involved in autophagy, lysosomal organization, and vesicle-mediated transport (**Figure 3C**).

To further substantiate the observation that COPD drives distinct transcriptional remodeling in males and females, we performed pathway-level analyses using Gene Set Enrichment Analysis (GSEA) as a complementary approach. GSEA was first performed on genes differentially expressed in the overall cohort (COPD vs control, both sexes combined), followed by sex-stratified analyses (male COPD vs male control; female COPD vs female control). GSEA revealed significant enrichment of interferon signaling, interferon alpha and gamma response, cytokine signaling in immune response, and inflammatory response pathways among the upregulated genes in COPD patients compared with controls (**Figure 3D**, COPD). Notably, these pathways were specifically enriched in male, but not female, patients when sex-disaggregated analysis was performed (**Figure 3D**; **Supplementary Table 4**).

To investigate whether the sex-specific transcriptional profiles identified in COPD neutrophils are pre-existing features also present in control donors or arise *de novo* in COPD patients, we compared male versus female neutrophils in both conditions.

Differential expression analysis revealed a substantial baseline sexual dimorphism, with 1,802 genes differentially expressed between male and female controls (adjusted p-value<0.05; **Supplementary Figure 3A**), and 3,857 genes differentially expressed between male and female COPD (**Supplementary Figure 3B**).

To identify the biological pathways underlying these differences, we performed GSEA analysis. In control donors, genes more expressed in males were significantly enriched for oxidative phosphorylation, whereas genes more expressed in females were enriched for apoptosis (**Supplementary Figure 3C**), indicating that sex-specific metabolic and cell-death programs are already established under physiological conditions.

We next examined sex-specific pathway enrichment in COPD patients. Consistent with controls, genes more expressed in male COPD patients compared to females were significantly enriched for oxidative phosphorylation, indicating preservation of this metabolic sex bias in disease (**Supplementary Figure 3D**). In addition, male-biased genes in COPD showed significant enrichment for inflammatory response, interferon-α response, and interferon-γ response pathways, highlighting the emergence of a strong immune activation signature specifically in males. No significantly enriched pathways were identified among genes more expressed in female COPD compared to males.

To distinguish between stable sex differences and disease-induced changes, we performed GSVA. The sex-biased pathways identified in controls—oxidative phosphorylation in males and apoptosis in females—showed comparable activity between COPD patients and controls (**Supplementary Figures 3E, F**), indicating that these represent stable, baseline sex-specific features that are not substantially modulated by disease. In contrast, interferon-α response, interferon-γ response, and inflammatory response pathways were significantly upregulated in male COPD patients compared to male controls (**Supplementary Figures 3G–I**), supporting the emergence of novel, disease-associated sex-specific signatures.

Collectively, these findings indicate that COPD does not simply amplify pre-existing sex differences but induces a partial transcriptional reprogramming of neutrophils.

Several studies have shown that donor characteristics, including age, smoking status, and disease severity, can influence immune cell transcriptional profiles. To exclude potential confounding effects, we performed a sex-stratified analysis of the clinical and demographic variables reported in **Supplementary Table 1**, confirming that these parameters did not differ between male and female COPD patients or controls (**Supplementary Table 2**). Although no sex-associated differences were identified at the clinical level, we further assessed whether sex-specific transcriptional signatures were associated with disease severity or smoking status. Using GSVA, we derived pathway activity scores for each patient, summarizing inflammatory and interferon (IFN-α and IFN-γ) responses in males, and lysosomal, lytic vacuole, autophagy, and macroautophagy processes in females. Correlation analyses revealed no significant associations between these pathway scores and age, disease severity, or smoking status in either males (**Supplementary Figure 4**) or females (**Supplementary Figure 5**).

### 3.3 Upstream inflammatory mediators driving sex-specific neutrophil responses in COPD

To identify factors underlying the sex-dependent transcriptional remodeling of neutrophils in COPD, we investigated potential upstream regulators using Ingenuity Pathway Analysis (IPA). This strategy analyzes, integrates, and interprets data from RNA-seq and identifies potential upstream molecules – such as transcription factors, cytokines, kinases, or drugs – that may be causing the observed expression changes.

**Figure 4:**
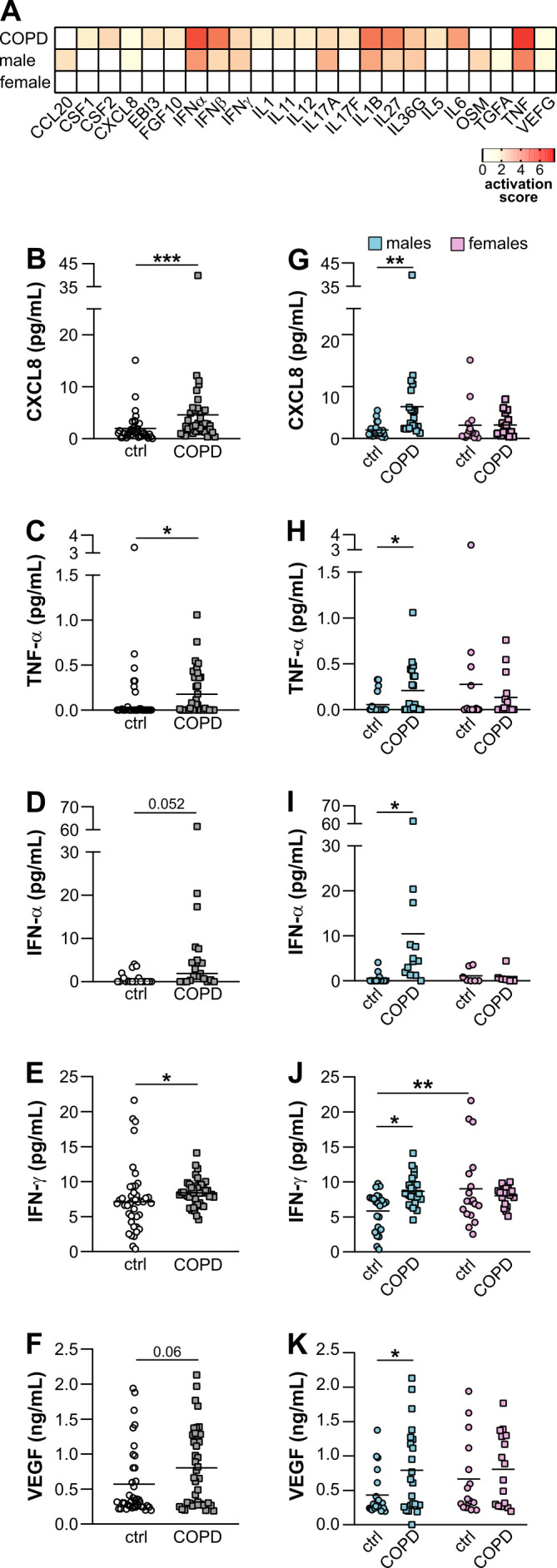
Sex-dependent inflammatory signaling in COPD. **(A)** Upstream regulator analysis performed using Ingenuity Pathway Analysis (IPA) on the COPD-associated signature and on DEGs identified in neutrophils from male and female COPD patients compared with sex-matched controls. Activation scores are reported according to the color scale indicated; non-significant predictions were set to 0. **(B–K)** Plasma levels of CXCL8, TNF-α, IFN-α, IFN-γ, and VEGF in COPD patients and control donors. Black lines indicate the mean value. *p-value < 0.05, **p-value < 0.01, ***p-value < 0.001, determined by Student’s t-test or Mann–Whitney test, as appropriate, or by two-way ANOVA followed by Tukey’s multiple comparison test.

IPA analysis identified chemokines, cytokines, interferons, and growth factors as putative upstream regulators of the transcriptional rewiring observed in male patients but not in females (**Figure 4A**). In order to validate IPA predicted upstream regulators, serum mediators were quantified by ELISA. According to IPA results, COPD patients had higher plasma levels of CXCL8 (COPD: 4.58 ± 1.05 pg/mL, ctrl: 1.97 ± 0.44 pg/mL) (**Figure 4B**), TNF-α (COPD: 0.18 ± 0.04 pg/mL, ctrl: 0.01 ± 0.09 pg/mL) (**Figure 4C**), IFN-α (COPD: 1.90 ± 1.20 pg/mL, ctrl: 0.03 ± 0.02 pg/mL) (**Figure 4D**), IFN-γ (COPD: 8.40 ± 0.28 pg/mL, ctrl: 7.10 ± 0.72 pg/mL) (**Figure 4E**), and VEGF (COPD: 0.80 ± 0.09 ng/mL, ctrl: 0.57 ± 0.08 ng/mL) (**Figure 4F**), but not of IL-1β and IL-17A (**Supplementary Figure 6**), compared to control donors. Importantly, increased plasma levels were observed exclusively in male patients upon sex-disaggregated analysis (**Figures 4G-K**). To further rule out the contribution of cytokine receptors expression to the transcriptional differences observed in neutrophils, we examined the mRNA levels of receptors for CXCL8 (CXCR1, and CXCR2), TNF-α (TNFR1, and TNFR2), IFN-α (IFNAR1, and IFNAR2), IFN-γ (IFNGR1, and IFNGR2), VEGF (FLT1), IL-1β (IL1R1), and IL-17A (IL17RA). No significant differences between COPD and controls were detected in either males or females (**Supplementary Figure 7**). Consistent with previous reports (19, 20), VEGFR2, VEGFR3, and IL17RC were not expressed in our RNA-seq data.

### 3.4 Epigenetic remodeling is associated with sex-specific transcriptional activation in COPD neutrophils

Given the sex-dependent differences in neutrophil transcriptional activation and cytokine milieu observed in COPD patients, we next explored whether epigenetic mechanisms could account for this differential responsiveness. In particular, we focused on histone 3 lysine 27 acetylation (H3K27ac), a hallmark of active enhancers and promoters associated with transcriptional activation (21).

**Figure 5:**
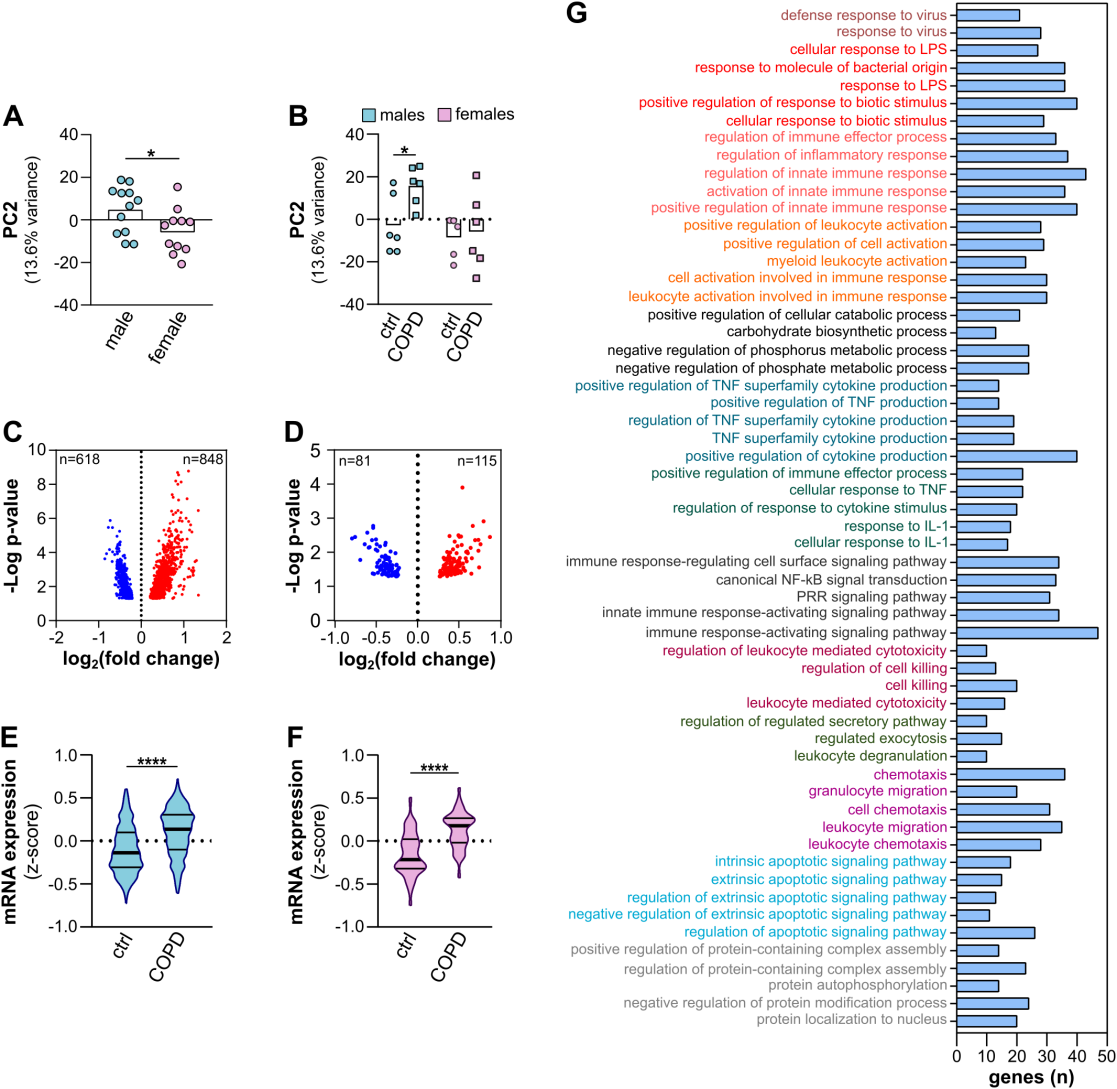
Sex influences the H3K27ac epigenetic landscape of neutrophils in COPD. PCA of the 500 most variable H3K27ac regions identified in neutrophils from COPD patients and control donors. **(A, B)** PC2 scores stratified by sex **(A)** and disease status **(B)**. Bars represent the median value. *p-value< 0.05, determined by Mann–Whitney test **(A)** or two-way ANOVA followed by Tukey’s multiple comparison test **(B)**. **(C, D)** Volcano plots showing differentially acetylated peaks in neutrophils from male **(C)** and female **(D)** COPD patients compared with controls (p-value< 0.05, Wald test). **(E, F)** Violin plot of the mRNA expression levels of genes associated with hyperacetylated peaks in males **(E)** and females **(F)**. ****p-value< 0.0001 according to the Mann-Whitney test. **(G)** GO term enrichment analysis of genes annotated to upregulated H3K27ac peaks in male COPD patients. Bars indicate the number of associated genes; related biological processes are shown in the same color.

H3K27ac profiles were generated from neutrophils purified from 23 donors (12 COPD patients: 6 males and 6 females; 11 controls: 6 males and 5 females) among those previously analyzed by RNA sequencing. Principal component analysis (PCA) of the 500 most variable acetylated regions was performed, and according to Elbow statistics (**Supplementary Figure 8A**), the first nine principal components (PCs) were examined. None of the PCs clearly separated patients from controls overall (**Supplementary Figures 3B–J**). However, when stratified by sex, PC2 distinguished males from females (**Figure 5A**) and, importantly, separated COPD patients from controls among males but not among females (**Figure 5B**). No significant sex-related differences were observed in the other PCs (**Supplementary Figures 8K–R**).

Sex-disaggregated differential analysis revealed 1,466 acetylated regions altered in male COPD patients compared to controls (**Figure 5C**), and 196 in females (**Figure 5D**). Among these, increased acetylation correlated positively with mRNA expression of associated genes in both males (**Figure 5E**) and females (**Figure 5F**). GO enrichment analysis of genes linked to the 848 hyperacetylated regions in males highlighted biological processes related to immune activation and inflammatory responses, including “response to virus,” “cytokine production,” “NF-κB signaling,” “myeloid leukocyte activation,” and “chemotaxis” (**Figure 5G**). In contrast, no significant enrichment was detected among the 115 most acetylated regions in females.

Together, these results demonstrate that COPD leads to extensive transcriptional and epigenetic rewiring in neutrophils primarily from male patients, characterized by activation of inflammatory and interferon pathways, remodeling of histone modifications at regulatory regions, and elevated levels of systemic inflammatory mediators.

## 4 Discussion

This study demonstrates that sex is a major determinant of neutrophil transcriptional and epigenetic remodeling in COPD, controlling both the magnitude of transcriptional changes and the immune pathways preferentially activated during disease. Unsupervised clustering and principal component analysis revealed that sex, rather than the COPD pathology itself, accounted for the largest variance in circulating neutrophil transcriptomes. This observation aligns with prior large-scale transcriptomic studies on circulating leukocytes of healthy subjects demonstrating pervasive sex-dependent differences in immune cell gene expression, including those of neutrophils, monocytes, and T cells (4).

Notably, sex-specific transcriptional programs were already evident in circulating neutrophils from the control cohort. Gene set enrichment analysis revealed baseline enrichment of oxidative phosphorylation pathway in males and apoptotic programs in females, indicating that sex-dependent metabolic and cell-death signatures are established under physiological conditions and remain unaltered in the context of COPD pathology. This contrasts with recent studies showing that neutrophils from young healthy females exhibit higher basal expression of interferon-stimulated genes and enhanced responsiveness to type I interferons compared to males (22, 23). However, these observations are not necessarily inconsistent. A key distinction lies in the age of the populations studied: Gupta and colleagues examined young adults (20–31 years) (26), whereas our cohort comprises older individuals (65–81 years). This difference is particularly relevant, as sex hormones are known to play a central role in shaping sex-specific immune responses (24). Indeed, the enhanced interferon responsiveness observed in female neutrophils has been attributed primarily to hormonal regulation rather than to differences in circulating interferon levels or differential expression of interferon receptors. In contrast, given the advanced age of our cohort, sex hormones are unlikely to be the principal driver of the differences observed in our study.

Despite baseline divergence, our analyses revealed that COPD triggers a shared core transcriptional program across sexes. Gene set variation analysis confirmed enrichment of the previously identified COPD-associated neutrophil signature (13) in both male and female patients relative to controls, although the magnitude of this enrichment was higher in males. This finding suggests that COPD activates similar pathways across sexes, but with different degrees of transcriptional engagement.

Moreover, GO term enrichment analyses of neutrophil transcriptomic profiles re-analyzed in a sex-disaggregated framework, revealed that both sexes exhibited alterations in biosynthetic and metabolic processes. This shared signature is consistent with previous reports identifying innate immune activation and metabolic remodeling as central features of COPD-associated neutrophil dysfunction (25, 26). Beyond these common pathways, however, marked sex-specific differences emerged in the functional programs engaged by neutrophils. Male COPD patients showed a preferential enrichment of inflammatory and antiviral defense pathways, including type I interferon signaling, cytokine-mediated responses, and broad immune activation pathways. Notably, these inflammatory and interferon-driven pathways were not observed as sex-biased features in healthy controls, indicating that they arise *de novo* in the context of disease rather than representing amplification of pre-existing dimorphism. Type I and II interferon signaling, a hallmark of antiviral and inflammatory responses, has been repeatedly implicated in COPD exacerbations and disease progression (11, 27). The male-biased enrichment of these pathways may reflect heightened systemic inflammatory tone.

In contrast, neutrophils from female COPD patients displayed preferential enrichment of autophagy- and vesicle-associated processes. This finding is of particular interest in the context of existing evidence suggesting that autophagy contributes to the regulation of neutrophil function during COPD pathogenesis. Previous studies have shown that induction of autophagy in neutrophils can promote apoptosis and accelerate disease progression through activation of the platelet-activating factor receptor, linking autophagic signaling directly to inflammatory tissue damage (28). In addition, COPD has been reported to enhance autophagic activity in neutrophils, thereby facilitating increased formation of neutrophil extracellular traps (NETs), a process known to exacerbate airway inflammation and tissue injury (29).

This dual pattern — shared yet differentially modulated — provides a unifying explanation for why COPD-associated gene signatures appear globally upregulated across all patients in sex-corrected analyses, yet yield statistically significant disease effects only in men when analyzed separately. More broadly, our data support a two-layer model of sexual dimorphism: a stable baseline layer comprising conserved metabolic and apoptotic programs, and a disease-inducible layer characterized by sex-specific activation of inflammatory pathways, particularly in males.

Integration of transcriptional data with epigenomic profiling together with circulating cytokine measurements supports the hypothesis that systemic inflammation contributes to the sex-dependent transcriptional rewiring observed in COPD. Epigenomic profiling identified widespread H3K27ac hyperacetylation at promoters and enhancers of immune genes in male COPD neutrophils, correlating with transcriptional upregulation of cytokine signaling, interferon response, and NF-κB pathways. In contrast, female neutrophils displayed limited acetylation changes and no significant enrichment of inflammatory terms. Additionally, plasma cytokine profiling revealed higher levels of chemokines (CXCL8), pro-inflammatory cytokines (TNF-α), and interferons in male patients, but not in females, mirroring the transcriptional enrichment of immune response pathways. These soluble mediators are well-known to contribute to neutrophil recruitment, oxidative stress, and tissue injury in COPD (30). This reinforces the concept that males experience a more pronounced systemic inflammatory burden, potentially linked to both environmental exposures (e.g., smoking intensity, comorbidities) and intrinsic immune differences. Such findings parallel epidemiological observations that men tend to exhibit more severe airflow limitation, greater neutrophilic inflammation, and faster decline in lung function (31), whereas women more frequently display small-airway disease and higher susceptibility to environmental triggers at lower exposure thresholds (32).

Sex-specific alterations in neutrophil transcriptional programs have also been reported in other inflammatory disorders. Recently, Wang et al. reported pronounced sex-related differences in circulating neutrophils from patients with Behçet’s uveitis (23). Consistent with our findings, neutrophils from male patients with Behçet’s uveitis exhibit an enhanced pro-inflammatory profile. In contrast, the type I interferon response was attenuated in this context. These observations, however, are not necessarily contradictory. Indeed, single-cell RNA sequencing analyses by Wang et al. demonstrated that the observed sex bias reflects both intrinsic transcriptional reprogramming and shifts in neutrophil subset composition between patients and healthy controls. Such compositional changes, in contrast, have not been reported in a single-cell RNA sequencing analysis in COPD (11), suggesting distinct underlying mechanisms. Furthermore, disease-specific differences in the systemic inflammatory milieu are likely to shape neutrophil transcriptional states. In COPD, we report elevated circulating levels of cytokines, chemokines, and both type I and type II interferons, contributing to broad neutrophil activation and reprogramming. Conversely, current evidence does not support a comparable increase in type I interferon levels in Behçet’s disease (33–35). This divergence in cytokine environments may account for the absence of an augmented type I IFN transcriptional signature in neutrophils from male patients with Behçet’s uveitis.

Methodologically, our results underscore the critical importance of considering sex not only as a covariate but also as a biological variable capable of interacting with disease effects. Conventional sex-corrected analyses, while valuable for identifying shared disease signatures, can obscure true biological interactions by removing sex-associated variance from disease-driven transcriptional responses. Conversely, fully disaggregated analyses reveal sex-specific regulatory programs but may underrepresent shared biology. Integrating both perspectives — as done here — offers a more complete understanding of how sex modulates disease-related molecular phenotypes.

From a translational standpoint, recognizing sex-dependent transcriptional architectures in COPD neutrophils has practical implications for biomarker discovery and therapeutic design. Drugs targeting interferon signaling, cytokine pathways, or autophagy may have differential efficacy across sexes, and sex-stratified analysis could enhance predictive precision in transcriptomic biomarkers of disease progression or treatment response.

Dysregulation of neutrophil phenotype and function is a central feature of COPD, not only in circulation but also within the lung microenvironment, including the walls and lumens of large and small airways. In these compartments, neutrophils actively contribute to tissue damage and chronic inflammation (36). However, while circulating neutrophils have been extensively studied, tissue-associated neutrophils remain less well characterized.

This gap is particularly relevant in the context of sex differences, as COPD manifests heterogeneously in the lung, encompassing phenotypes such as chronic bronchitis and emphysema, the latter being more prevalent in males than in females (2). The absence of lung biopsies or bronchoalveolar lavage (BAL) samples in our study precluded the investigation of how local microenvironmental cues may differentially shape neutrophil behavior in males and females. Addressing this limitation will be essential, as a better understanding of sex-related differences in tissue-resident neutrophils across distinct COPD phenotypes could provide critical insights into disease mechanisms and refine our understanding of neutrophil-driven pathology in COPD.

In summary, our study delineates a dual model of COPD-associated neutrophil transcriptional remodeling: a shared core program of inflammatory and metabolic activation present in both sexes, and sex-specific amplifications that modulate the magnitude and functional focus of this program.

These findings underscore the need to incorporate sex as a fundamental biological axis in mechanistic and translational research, paving the way for sex-aware strategies in COPD diagnosis, prognosis, and therapy.

## Data Availability

RNA sequencing data are publicly available in the GEO database (accession number GSE278400). ChIP sequencing data will be available in the GEO database after publication of the study.
