# Supplementary material for: Sex-dependent transcriptional and epigenetic regulation of neutrophil inflammatory programs in COPD
Source: Front Immunol. 2026 May 28;17:1824596. doi: 10.3389/fimmu.2026.1824596 (PMC13252782; doi:10.3389/fimmu.2026.1824596)
Supplement: Supplementary file 1 [file DataSheet1.docx]

Supplementary Material

Sex Shapes Neutrophil Transcriptional and Epigenetic Programs in COPD

**Barbara Mariotti^1^, Sara Gasperini^1^, Chiara Bracaglia^1^, Carlo Frigenti^1^, Giulia Sartori^2^, Claudia di Chiara^2^, Francesca Sangiovanni^2^, Ernesto Crisafulli^2^, and Flavia Bazzoni^1*^.**

^1^Department of Medicine, Division of General Pathology, University of Verona, Verona, Italy

^2^ Department of Medicine, Respiratory Medicine Unit, University of Verona, and Azienda Ospedaliera Universitaria Integrata of Verona, Verona, Italy

*** Correspondence:**Department of Medicine

Division of General Pathology

University of Verona, Verona, Italy

Strada Le Grazie 8, 37134 Verona Italy

Email: [flavia.bazzoni@univr.it](mailto:flavia.bazzoni@univr.it)

# Figure S1


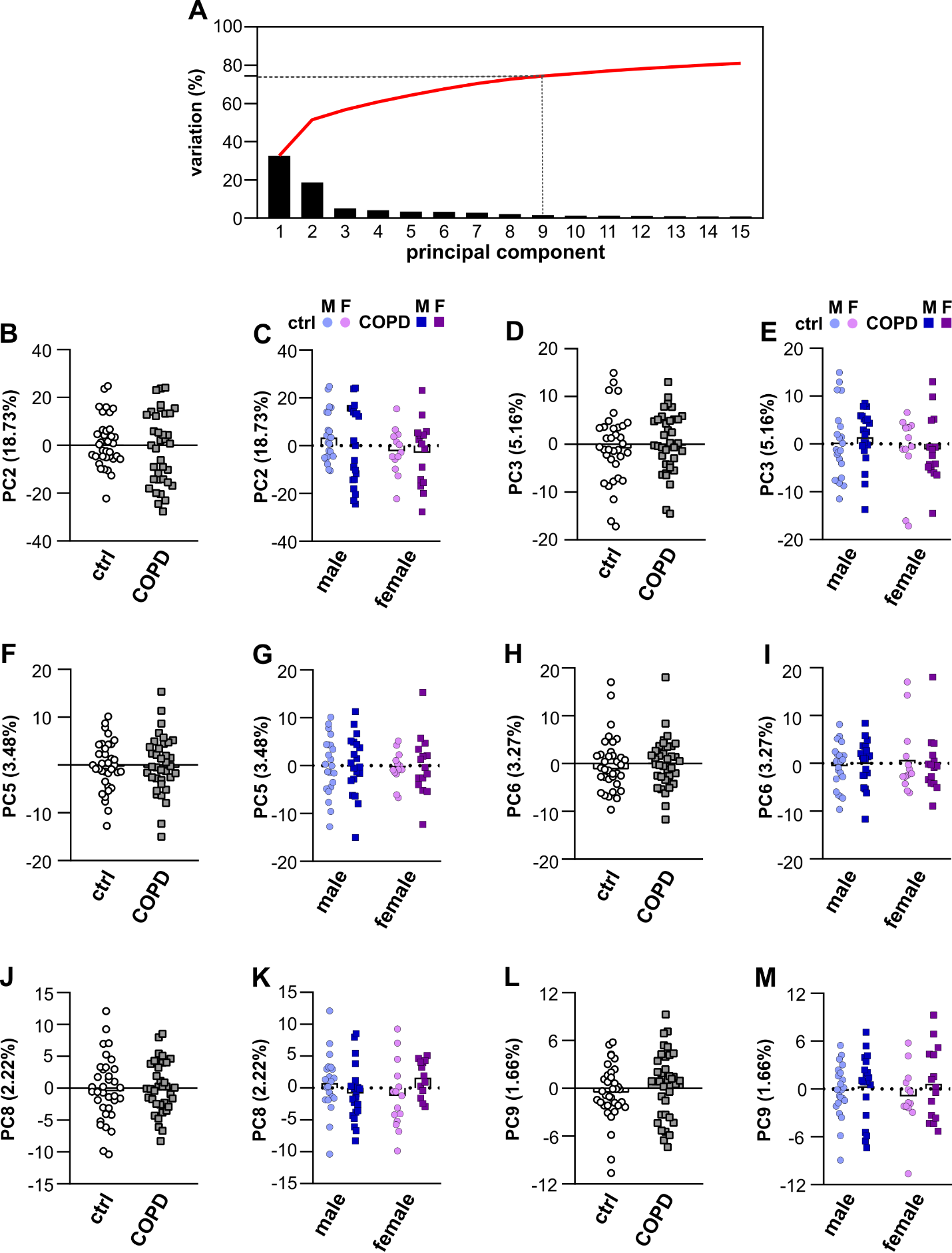


Figure S1 Principal component analysis (PCA) of the transcriptional profile of circulating neutrophils

(A) Scree plot of the PCA of the transcriptomic profile of neutrophils from COPD patients and control donors. The red line indicates the cumulative percentage of explained variance, and dotted lines denote the last principal component selected based on Elbow statistics. (B-M) PC2, PC3, PC5, PC6, PC8, and PC9 scores in COPD patients and control donors. Male donors are shown in blue and female donors in pink (C, E, G, H, I, K, M). Bars represent median values. The percentage of variance explained by each principal component is reported.

# Figure S2


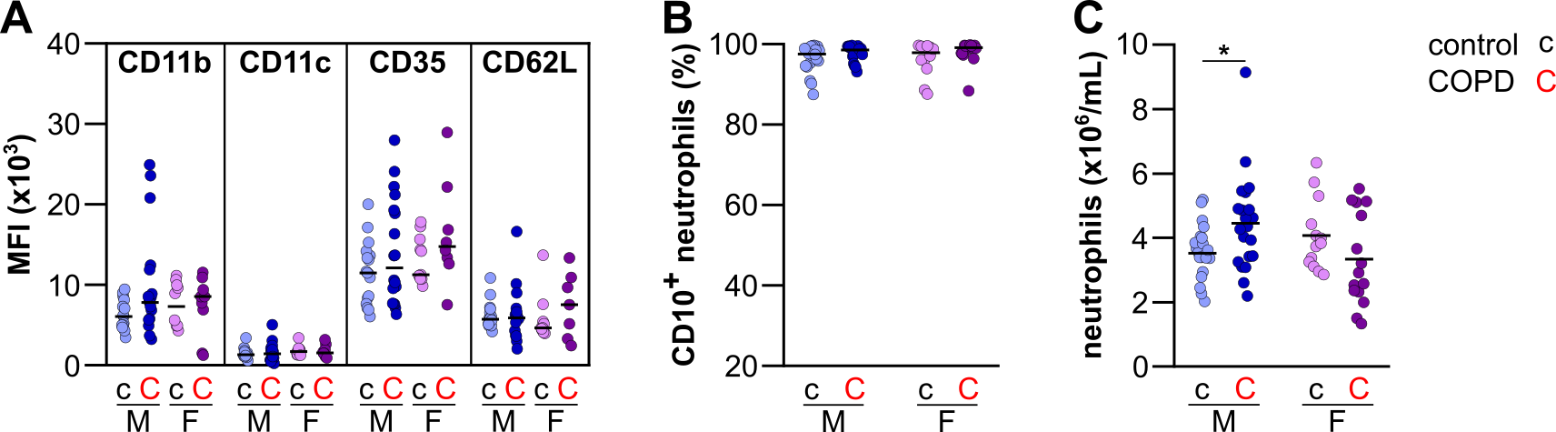


Figure S2 Activation, maturation state, and number of circulating neutrophils in male and female COPD patients and control donors

(A) FACS analysis of CD11b, CD11c, CD35, and CD62L expression in neutrophils from male and female COPD patients and control donors. Data are shown as Mean Fluorescent Intensity (MFI, CD11b) or delta MFI calculated as previously described (1). (B) Percentage of mature CD10^+^ neutrophils in male and female COPD patients and controls as determined by FACS analysis. (C) Neutrophil count in whole blood of male and female COPD patients and controls. Data are shown as millions of cells per mL of blood (×10^6^/mL). Median values are shown as black lines. c: control donors; C: COPD patients; M: male; F: female. * p-value < 0.05 according to Two-way ANOVA followed by Sidak’s multiple comparison test.

# Figure S3


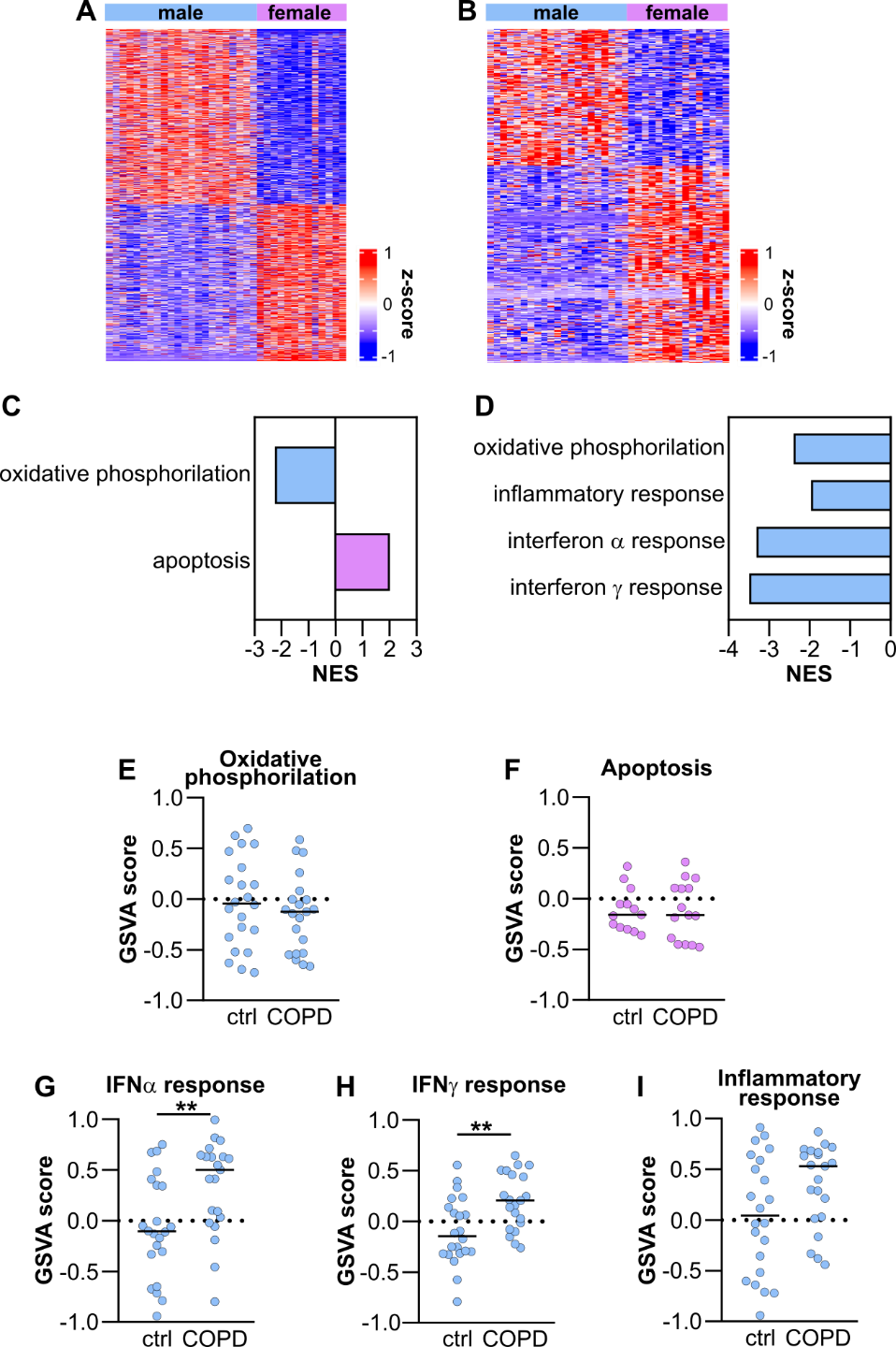


Figure S3 Characterization of sex-associated transcriptional differences in controls and COPD patients

Heatmaps of differentially expressed genes in neutrophils from male control donors compared to females (A) and male COPD patients compared to females (B). Heatmaps show the z-score of the Fragment per Million mapped reads (FPM). (C, D) Results of the Gene Set Enrichment Analysis (GSEA) of differentially expressed genes in controls (C) and in COPD patients (D). The normalized enrichment score (NES) of significantly (FDR < 0.05) enriched hallmark gene sets was depicted. Blue bars: hallmarks enriched in males; pink bars: hallmarks enriched in females. (E-I) Gene Set Variation Analysis (GSVA) of the hallmark of oxidative phosphorylation (E), IFN-α response (G), IFN-γ response (H), and inflammatory response (I) in males and of the hallmark of apoptosis in females (F). ** p-value < 0.01 according to the Mann-Whitney test.

# Figure S4


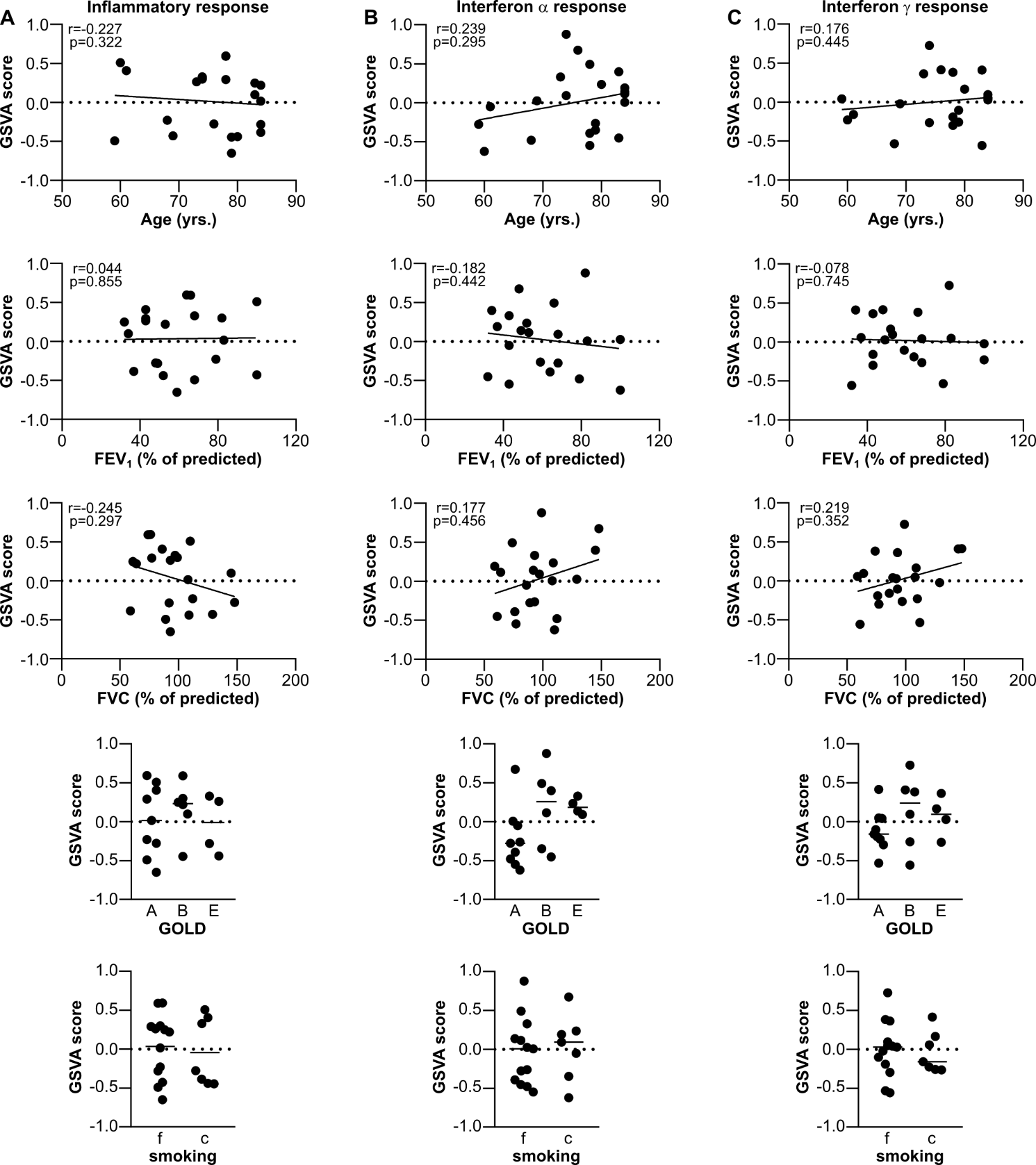


Figure S4 Correlation analysis between age, disease severity, smoking, and male-specific pathways

The expression of genes from the inflammatory response (A), interferon α response (B), and interferon γ response (B) pathways was correlated with age, FEV_1_ (% of predicted), FVC (% of predicted), GOLD stage, and smoking status in male patients. Expression of genes from the selected pathway in each patient was summarized using GSVA and reported as a GSVA score. Spearman correlation coefficient (r) and p-value were reported. f: former smoker; c: current smoker.

# Figure S5


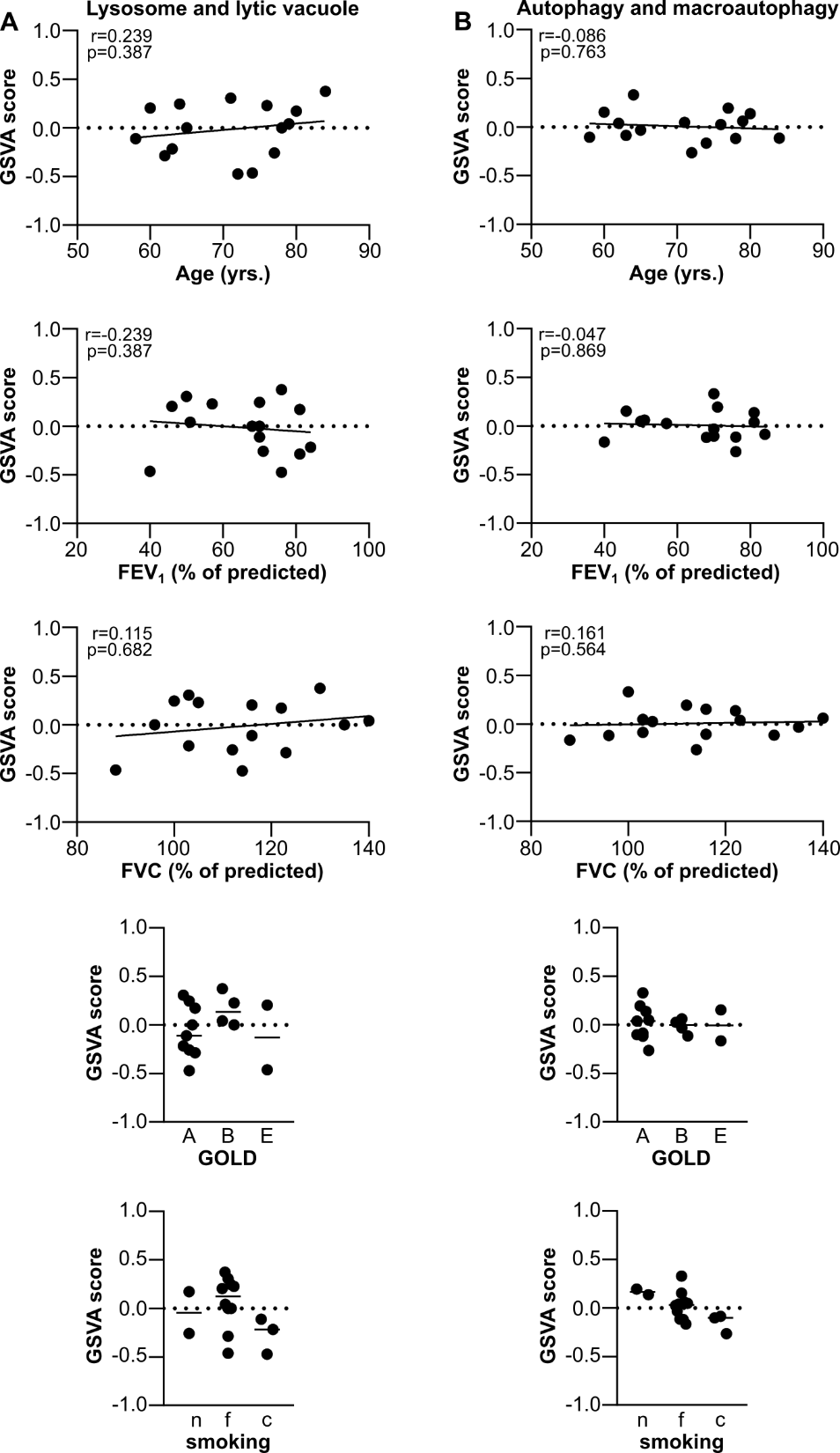


Figure S5 Correlation analysis between age, disease severity, smoking, and female-specific pathways

The expression of genes associated with lysosome and lytic vacuole (A) and autophagy and macroautophagy (B) biological processes was correlated with age, FEV_1_ (% of predicted), FVC (% of predicted), GOLD stage, and smoking status in female patients. Expression of genes from the selected pathway in each patient was summarized using GSVA and reported as a GSVA score. Spearman correlation coefficient (r) and p-value were reported. n: never smoker, f: former smoker; c: current smoker.

# Figure S6


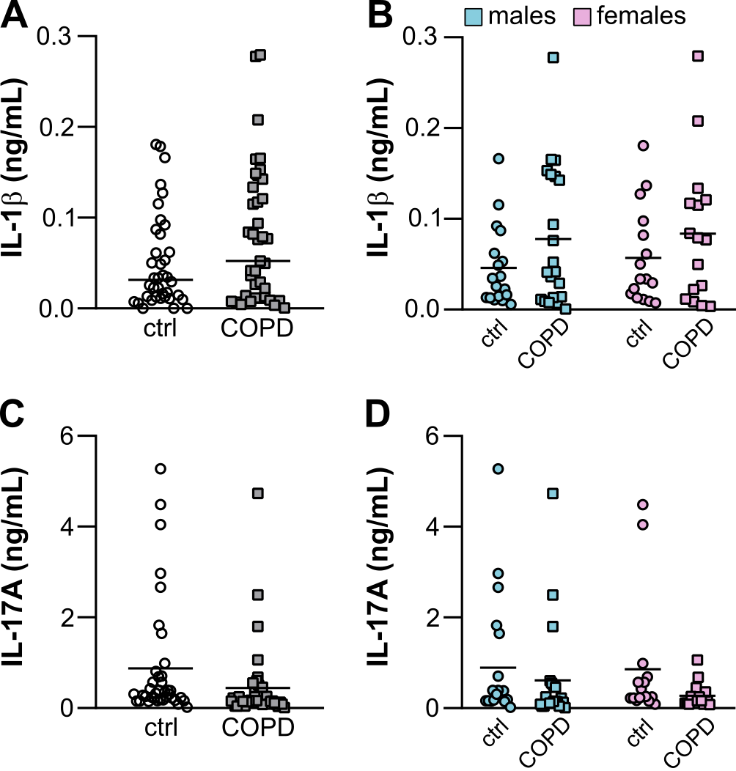


Figure S6 Plasma levels of IL-1β and IL-17A in COPD

# Plasma concentrations of (A, B) IL-1β and (C, D) IL-17A in COPD patients and control donors. Black lines indicate the mean value.Figure S7


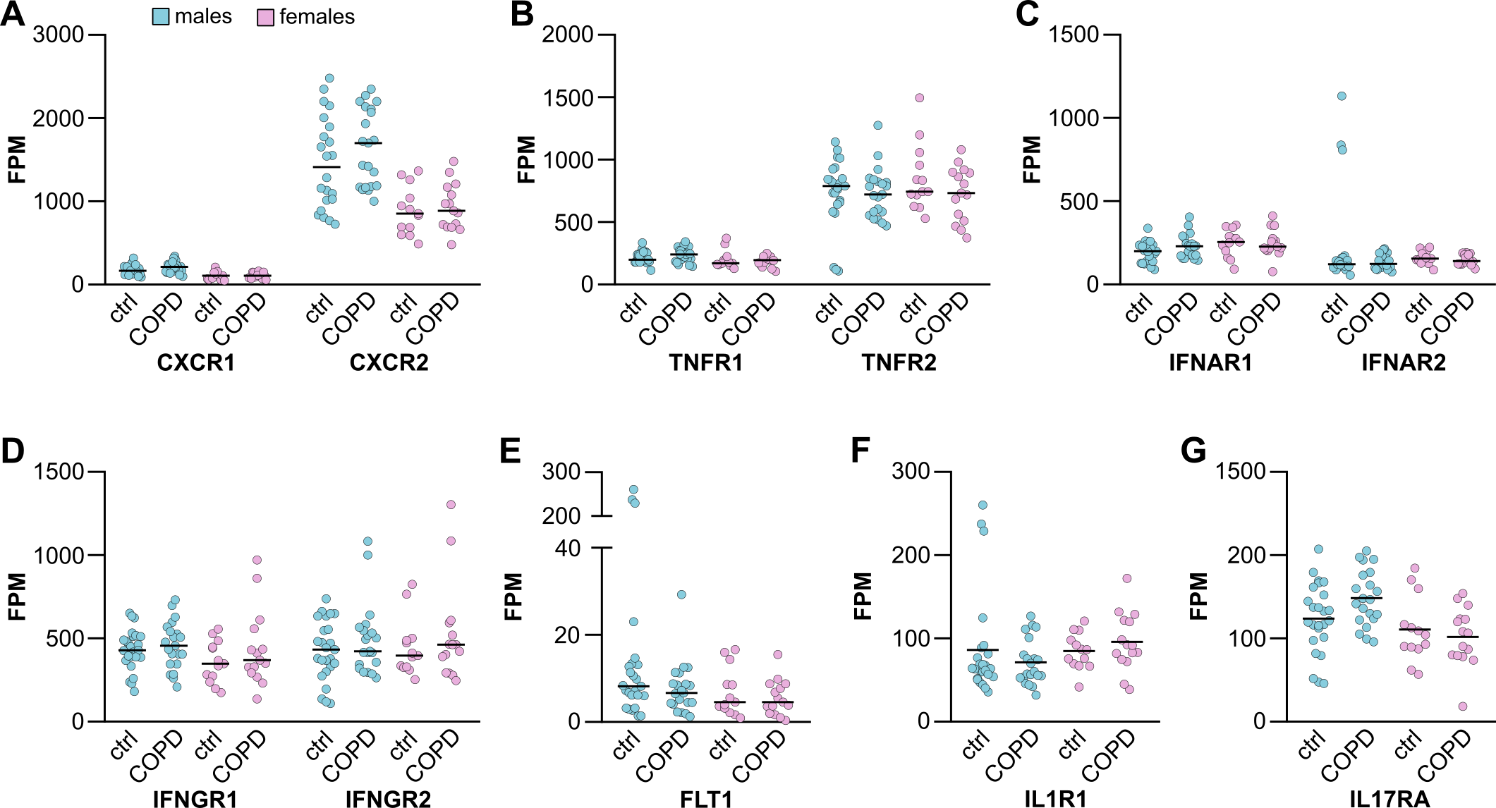


Figure S7 Expression levels of cytokine receptors in circulating neutrophils

Expression of (A) CXCR1, CXCR2, (B) TNFR1, TNFR2, (C) IFNAR1, IFNAR2, (D) IFNGR1, IFNGR2, (E) FLT1, (F) IL1R1, and (G) IL17RA in neutrophils from COPD patients and control donors. Light blue: males, pink: females. Black lines indicate the mean value. Data are shown as Fragments per Million mapped reads (FPM) determined by RNA-seq analysis.

# Figure S8

#
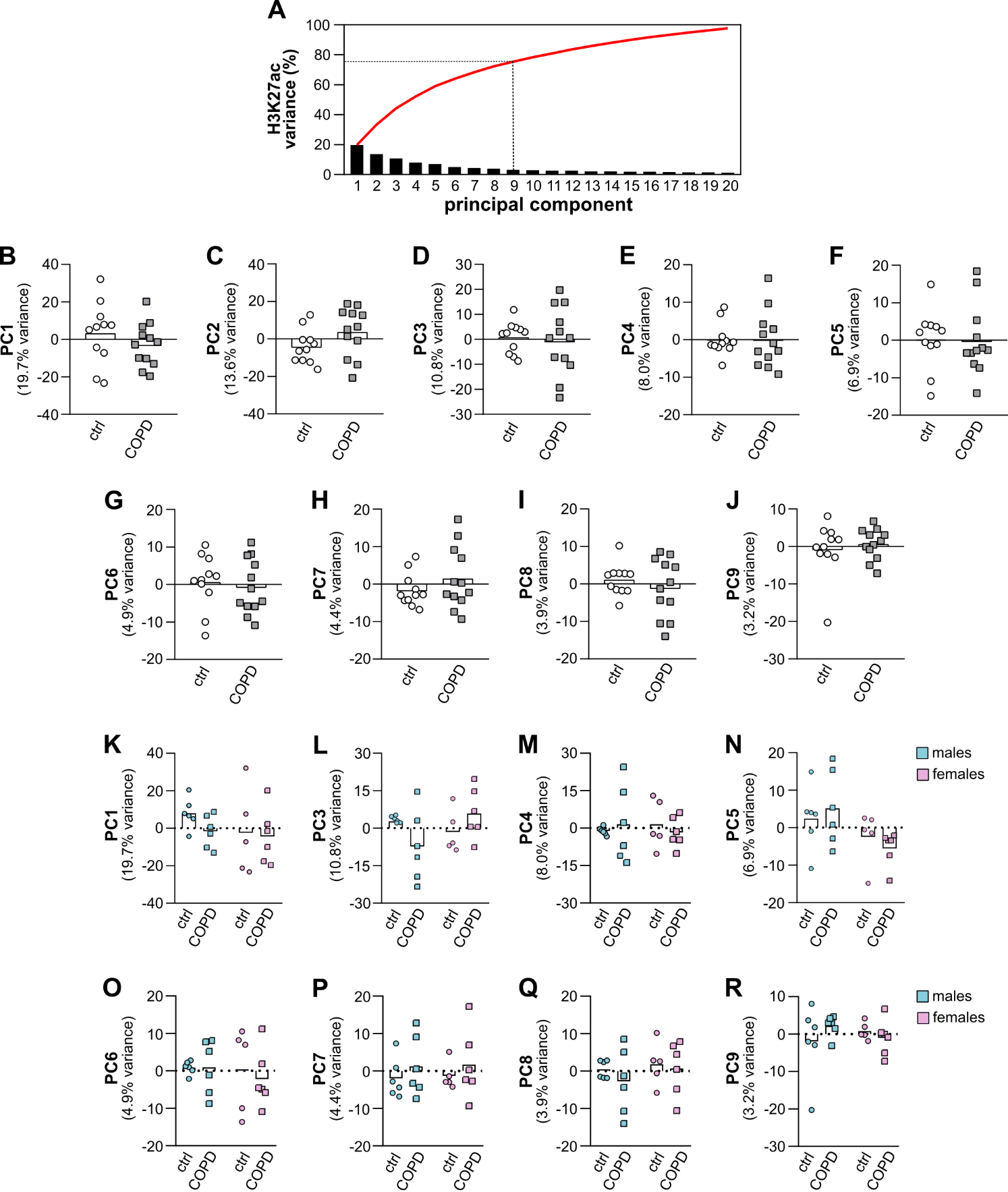


Figure S8 Principal component analysis of the H3K27ac profile of circulating neutrophils

(A) Scree plot of PCA performed on H3K27ac profiles from neutrophils of COPD patients and control donors. The red line indicates cumulative variance, and dotted lines mark the last principal component selected using Elbow statistics. (B–R) PC1–PC9 scores in COPD patients and control donors. Male donors are shown in blue and female donors in pink (K–R). Bars represent the median value, and the percentage of variance explained by each principal component is reported.

# Table S1 Clinical and demographic data for recruited COPD and control subjects

|  | **Control donors** | | **COPD patients** | |  |
| --- | --- | --- | --- | --- | --- |
|  | **n** | **Median (Q1-Q3)** | **n** | **Median (Q1-Q3)** | **p-value** |
| **Sex (M/F)** | 35 | 22/13 | 36 | 21/15 | 0.809 ^χ^ |
| **Age** | 35 | 78.0 (65.0-81.0) | 36 | 76.0 (66.8-79.8) | 0.468 ^$^ |
| **BMI** | 27 | 25.6 (23.7-27.7) | 29 | 26.5 (24.0-29.5) | 0.485 ^t^ |
| **Smoker (n/f/c)** | 35 | 24/9/2 | 34 | 2/22/10 | **<0.0001 ^χ^** |
| **CC Index*** | 24 | 2 (0-3) | 35 | 1 (1-3) | 0.835 ^$^ |
| **FEV_1_, L** | 21 | 2.7 (2.1-3.0) | 35 | 1.2 (0.9-1.8) | **<0.0001 ^$^** |
| **FEV_1_, % of predicted** | 21 | 110 (102-129) | 35 | 66 (48-76) | **<0.0001 ^t^** |
| **FVC, L** | 21 | 3.4 (2.5-3.6) | 35 | 2.6 (1.9-3.4) | 0.058 ^$^ |
| **FVC, % of predicted** | 21 | 108 (99-120) | 35 | 103 (89-116) | 0.238 ^t^ |
| **FEV_1_/FVC (%)** | 21 | 77 (76-79) | 35 | 51 (40-60) | **<0.0001 ^t^** |
| **mMRC** |  |  | 34 | 1 (1-2) |  |
| **GOLD 2023 (A/B/E)** |  |  | 34 | 18/10/6 |  |

Values reported indicate the number (n) of subjects and/or the median for each parameter (interquartile range, if not otherwise indicated). *COPD pathology was not included in Charlson index; t – Student t-test, $ – Mann-Whitney test, χ – Fisher’s exact test; significant (p-value<0.05) differences are in bold. **Abbreviations:** BMI, body mass index; n/f/c, never/former/current; CC index, Charlson Comorbidity index; COPD, chronic obstructive pulmonary disease; FEV_1_, forced expiratory volume in 1 second; FVC, forced vital capacity; GOLD, The Global Initiative for Obstructive Lung Disease.

| **Males** | | | | **Females** | | | |  |  |
| --- | --- | --- | --- | --- | --- | --- | --- | --- | --- |
| **Controls** | | **COPD** | | **Controls** | | **COPD** | |  |  |
| **n** | **Median (Q1-Q3)** | **n** | **Median (Q1-Q3)** | **n** | **Median (Q1-Q3)** | **n** | **Median (Q1-Q3)** | **p-value** |  |
| **Age** | 22 | 79  (75-81) | 21 | 78  (71-83) | 13 | 75  (65-79) | 15 | 72  (63-78) | 0.096 ^k^ |
| **BMI** | 17 | 24.8  (23.6-28.5) | 14 | 26.3  (24.2-29.1) | 10 | 26.2  (23.4-28.4) | 15 | 28.0 (22.1-30.0) | 0.924 ^w^ |
| **Smoker**  **(n/f/c)** | 22 | 17/5/0 | 19 | 0/12/7 | 13 | 7/4/2 | 15 | 2/10/3 | Ctrl:  0.120^χ^  COPD:  0.187^χ^ |
| **CC index*** | 15 | 1 (0-3) | 20 | 2 (1-3) | 9 | 2 (1-3) | 15 | 1 (0-2) | 0.231 ^k^ |
| **FEV_1_**  **% of predicted** | 13 | 110  (100-134) | 20 | 56  (43-76) | 8 | 110  (103-128) | 15 | 70  (51-76) | Ctrl:  0.968^h^  COPD:  0.592  ^h^ |
| **FVC**  **% of predicted** | 13 | 104  (96-119) | 20 | 93  (76-110) | 8 | 110  (105-127) | 15 | 114  (103-123) | Ctrl:  0.601^h^  COPD:  0.060 ^h^ |
| **FEV_1_/FVC%** | 13 | 77  (74-82) | 20 | 50  (39-62) | 8 | 78  (76-79) | 15 | 51  (40-56) | Ctrl:  0.961^h^  COPD:  0.944 ^h^ |
| **mMRC** |  |  | 19 | 1 (1-3) |  |  | 15 | 1 (1-2) | 0.208^$^ |
| **GOLD 2023 (A/B/E)** |  |  |  | 9/6/4 |  |  |  | 9/4/2 | 0.739 ^χ^ |

# Table S2 Clinical and demographic data for recruited COPD and control subjects stratified by sex

Values reported indicate the number (n) of subjects and/or the median for each parameter (interquartile range, if not otherwise indicated). *COPD pathology was not included in Charlson index; ^k^ – Kruskall-Wallis test, ^$^ – Mann-Whitney test, ^w^ – one-way ANOVA, ^h^ –Holm-Sidak’s multiple comparisons test, ^χ^ – Chi square test. **Abbreviations:** BMI, body mass index; n/f/c, never/former/current; CC index, Charlson Comorbidity index; COPD, chronic obstructive pulmonary disease; FEV_1_, forced expiratory volume in 1 second; FVC, forced vital capacity, GOLD, The Global Initiative for Obstructive Lung Disease.

# Table S3 Genes composing the COPD signature

Table S3 is reported in a separate Excel file.

# Table S4 Results of the GSEA

|  |  | **COPD** | | | **male** | | | **female** | | |
| --- | --- | --- | --- | --- | --- | --- | --- | --- | --- | --- |
| **Gene Set** | | **Size** | **NES** | **FDR** | **Size** | **NES** | **FDR** | **Size** | **NES** | **FDR** |
| **Hallmark** | **Interferon γ response** | 55 | 3.00 | 0.000 | 22 | 2.54 | 0.001 | ND | ND | ND |
|  | **Interferon α response** | 39 | 2.63 | 0.001 | 12 | 2.38 | 0.000 | ND | ND | ND |
|  | **Inflammatory response** | 18 | 1.63 | 0.053 | 21 | 1.93 | 0.023 | ND | ND | ND |
| **Reactome** | **Interferon signalling** | 48 | 2.39 | 0.004 | 22 | 2.37 | 0.003 | 6 | -0.39 | 0.982 |
|  | **Antiviral mechanism by IFN-stimulated genes** | 20 | 0.87 | 0.949 | 12 | 2.03 | 0.028 | ND | ND | ND |
|  | **Interferon γ signalling** | 22 | 2.03 | 0.020 | 11 | 1.92 | 0.050 | ND | ND | ND |
|  | **Cytokine signalling in immune system** | 79 | 2.25 | 0.005 | 59 | 1.92 | 0.054 | 19 | -0.86 | 1.000 |

**Abbreviations:** ES, enrichment score; FDR, false discovery rate; IFN, interferon; ND, not detected; NES, normalized enrichment score; TLR, Toll-Like Receptor.
